# Forced degradation studies of medroxyprogesterone acetate injectable suspensions (150 mg/ml) with implementation of HPLC, mass spectrometry, and QSAR techniques

**DOI:** 10.1016/j.jpba.2020.113352

**Published:** 2020-08-05

**Authors:** David Jenkins, Christopher L. Harmon, Xiao Jia, Allen Kesselring, Danielle Hatcher, Katie Grayson, Jennifer Ayres

**Affiliations:** a Product Quality and Compliance, FHI 360, 2810 Meridian Parkway, Suite 160, Durham, NC 27713, USA; b Product Development and Introduction, FHI 360, 359 Blackwell Street, Suite 200, Durham, NC 27701, USA; c EKG Life Science Solutions, 4633 World Parkway Circle, St. Louis, MO 63134, USA

**Keywords:** Medroxyprogesterone acetate, Forced degradation, HPLC-MS/MS, Quantitative structure activity relationships, Toxicity risk

## Abstract

Medroxyprogesterone acetate (MPA) injectable products are a key commodity for reproductive health and are available in the global market from a variety of manufacturing sources. Depending on the climatic zone conditions of the destination country for product use, MPA injectables are at risk of exposure to adverse transport and storage conditions. Analytical methods are available that quantify impurity levels in MPA and MPA injectable products, but minimal information is publicly available on the source of impurity and degradation product generation or the safety risk of these compounds. Forced degradation studies were conducted on MPA and MPA injectables to gain a better understanding of potential sources of impurities and degradation products. Furthermore, QSAR analysis was conducted to assess the toxicity risk of known impurities. More impurities were generated under acidic, basic, light, and oxidative forced degradation conditions relative to thermal degradation, however thermal exposure is the most likely adverse condition to be experienced by these products. Even if impurities are present in MPA injectables, QSAR analysis found that known impurities for MPA are apparently no more of a safety risk than MPA.

## Highlights

• Forced degradation of medroxyprogesterone acetate injectable contraceptives.
• Analysis of impurities and assessment of toxicity.
• Possible field exposure to environmental conditions and resulting safety risks.

## 1 Introduction

For global public health initiatives, various donors of reproductive health commodities, such as the United States Agency for International Development [1] and United Nations Population Fund [2], are continually trying to source a wide array of pharmaceuticals and medical devices to meet the needs of various country programs for family planning. For reproductive health pharmaceuticals, a range of manufacturing sources are available for procurement from many different countries of origin. The World Health Organization’s (WHO) prequalification program [3] serves as a helpful resource commonly utilized by donors when searching for product sources of reputable quality for a variety of health indications (i.e., HIV, malaria, tuberculosis, and reproductive health). Although the list is not exhaustive of the full range of suppliers in the global market, WHO’s prequalified list comprises of a range of over 40 reproductive health products, such as (a) tablets with desogestrel / ethinyl estradiol, levonorgestrel / ethinyl estradiol, levonorgestrel, norethisterone, mifepristone or misoprostol, (b) implants containing etonogestrel or levonorgestrel, or (c) injectables with medroxyprogesterone acetate, norethisterone enanthate, magnesium sulfate, or oxytocin.

Depot medroxyprogesterone acetate injections (DMPA-IM, 150 mg/mL) delivered through intramuscular injection can provide contraceptive protection for approximately 12 weeks (∼3 months) [4]. DMPA-IM has been a key product in reproductive health programs and procurement projections are estimated at a global level of over 150 million vials annually through 2020 [5]. Depending on the product(s) utilized within global public health programs, tropical conditions (i.e., zone IVa - 30 °C/65 %RH or zone IVb – 30 °C/75 %RH [6]) that the products may be exposed to during transit or storage may be stressful enough to cause various levels of deterioration. Many reproductive health products have been prequalified by the WHO with expiration dates based on a 30 °C maximum storage temperature, however several tablets and injectables have listed storage conditions not to exceed 25 °C [3]. Currently, three DMPA-IM (150 mg/mL) products have achieved WHO prequalification status, where one product is listed with a three-year shelf life (with storage at or below 30 °C), one has a two-year shelf life (with storage at or below 25 °C), and the other is listed with a five-year shelf life (with storage at or below 25 °C) [3].

For medroxyprogesterone acetate (MPA), methods are available for characterizing impurities and degradation products (i.e., related substances) in active pharmaceutical ingredient (API) [7,8] and injectable products [9,10]. More options are available for specific identifications and quantifications of impurities for API [7,8], with injectable products having fewer options for specific impurities. For injectable products, specific methods are available for impurity F (4,5-dihydromedroxyprogesterone acetate) [9], while the International Pharmacopoeia method can characterize several specific impurities [10]. These methods provide limits for the respective impurities, but information on the source of the impurity / degradation product (i.e., responsible degradation conditions or production impurity), or the basis of the limits is not readily available.

In this study, four medroxyprogesterone acetate injectable products (150 mg MPA/mL) from different manufacturers were exposed to a series of forced degradation conditions with subsequent high-performance liquid chromatography / tandem mass spectrometry (HPLC - MS / MS) to further characterize observed impurities. Furthermore, as toxicity data for MPA impurities are generally not publicly available, quantitative structure activity relationship (QSAR) analysis was conducted on impurities with assignable chemical structures to assess potential safety and toxicity risks. The overall goal of this work was to provide more insight regarding impurities and degradation products that can be generated in DMPA-IM under different forced degradation conditions and assess potential safety risks that may exist with different impurities.

## 2 Material and methods

### 2.1 Chemicals

**Fig. 1:**
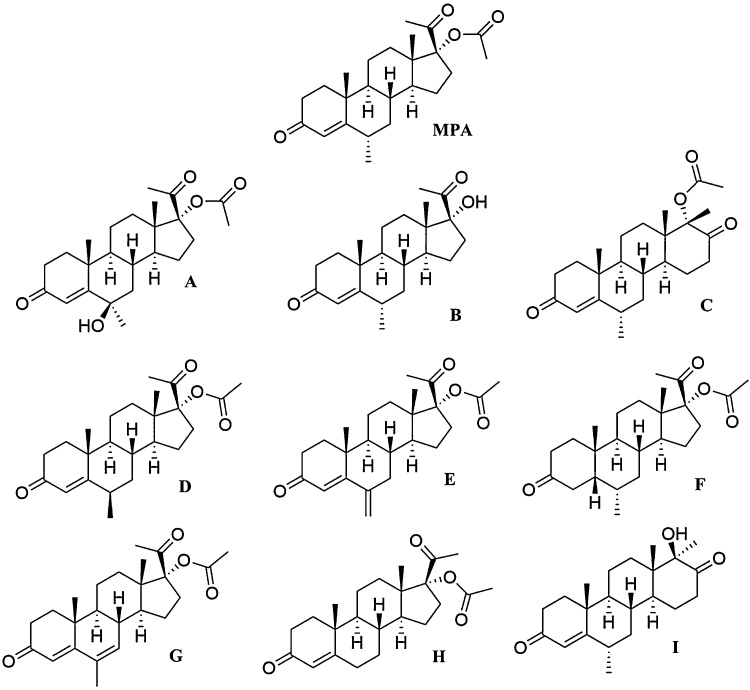
Chemical structures for medroxyprogesterone acetate (MPA) and its known impurities [8]. A - 6-hydroxymedroxyprogesterone acetate (6β-hydroxy-6-methyl-3,20-dioxopregn-4-en-17-yl acetate); B – medroxyprogesterone (17-hydroxy-6α-methylpregn-4-ene-3,20-dione); C - 6α,17α-dimethyl-3,17-dioxo-d-homoandrost-4-en-17α-yl acetate; D - 6-epimedroxyprogesterone acetate (6β-methyl-3,20-dioxopregn-4-en-17-yl acetate); E - 6-methylenehydroxyprogesterone acetate (6-methylidene-3,20-dioxopregn-4-en-17-yl acetate); F - 4,5-dihydromedroxyprogesterone acetate (6α-methyl-3,20-dioxo-5β-pregn-17-yl acetate); G - megestrol acetate (6-methyl-3,20-dioxopregna-4,6-dien-17-yl acetate); H - hydroxyprogesterone acetate (3,20-dioxopregn-4-en-17-yl acetate); **I** - 17β-hydroxy-6α,17α-dimethyl-d-homoandrost-4-en-3,17-dione.

Reagent grade quinine monohydrochloride dihydrate, sodium carbonate, and sodium hydroxide (1.0 N) and HPLC grade tetrahydrofuran and acetonitrile were purchased from Sigma Aldrich (St Louis, Missouri, USA). HPLC grade tetrahydrofuran (stabilized with ∼0.025 % 2,6-di-tert-butyl-4-methylphenol) and reagent grade hydrochloric acid solution (0.5 M) and hydrogen peroxide (30 % w/w) were purchased from Fluka (Mexico City, Mexico). Ultrapure water (18 megohm-cm) was obtained from a Millipore Direct-Q3 water purification system (Burlington, Massachusetts, USA). Medroxyprogesterone acetate for system suitability CRS (contains impurities A, B, C, D, E, G, I) and medroxyprogesterone acetate for peak identification (containing impurity F) were obtained from the European Pharmacopoeia through the European Directorate for the Quality of Medicines and Healthcare (EDQM, Strausbourg, France). Medroxyprogesterone acetate reference standard was obtained from the United States Pharmacopeia (USP, Rockwell, Maryland, USA). Fig. 1 provides the chemical structures for MPA and known impurities A–I.

### 2.2 Test materials

MPA powder was purchased from Sigma Aldrich (labeled as 100 % purity, St. Louis, Missouri, USA). Various sources of MPA injectable suspensions (150 mg/mL MPA) finished product were used for this study. The Greenstone® brand MPA injectable suspension, USP (Greenstone LLC, Peapack, NJ, USA) was purchased (7 vials from 1 lot) from a US pharmacy for research purposes. Depo-Provera® (Pfizer, Puurs, Belgium) was obtained (12 vials from each of 3 lots) as a donation through the USAID│DELIVER Project (http://deliver.jsi.com). Petogen® was obtained (10 vials from each of 3 lots) as a donation from Helm AG (by Fresenius Kabi Manufacturing, SA (Pty) Ltd. Port Elizabeth, South Africa). FAMY DEPO® was obtained (10 vials from each of 3 lots) as a donation from Mylan Laboratories Limited (by Jai Pharma Ltd., Ahmedabad, India). Results will not be provided with linkages to product brand names but will rather be labeled with samples codes (123, 183, 789, and 890) to protect manufacturer confidentiality.

### 2.3 Sample preparations for forced degradation conditions

For each of the following forced degradation conditions (light, heat, humidity, acid, base, and oxidative) [11,12], all MPA powder sample preparations were prepared in duplicate, where all finished product sample preparations were prepared from single vials for each of the lots available per finished product type.

#### 2.3.1 Controls (ambient conditions)

For MPA powder or resuspended finished product, 100 mg or 1 mL (respectively) was transferred to a 250 mL volumetric flask. Approximately 200 mL of diluent (50 % (v/v) acetonitrile in water) was added, and the mixture was sonicated for several minutes until the contents were dissolved. The flask was subsequently diluted to volume with diluent and mixed. Control samples were freshly prepared at the time of analysis of the other forced degradation samples.

#### 2.3.2 Light degradation

A 2% quinine solution was prepared by weighing 500 mg of quinine monohydrochloride dihydrate into a 25 mL volumetric flask. Approximately 15 mL of water was added, and the mixture was sonicated until the powder was dissolved. The flask was diluted to volume with water and mixed.

For MPA powder samples, 100 mg of sample was transferred into a petri dish and spread into a thin layer. For finished product samples, 1 mL of resuspended sample was transferred into a 20 mL scintillation vial and capped. Into two separate ampules, 10 mL of 2% quinine was transferred and sealed (hermetically with a torch). One ampule was wrapped in aluminum foil and the other ampule remained uncovered.

The samples and the two ampules were placed in a photostability chamber (Custom fabricated photochamber achieving ICH Q1B Option 2 [12] with twelve cool white fluorescent and four near UV fluorescent lamps, St Louis Missouri, USA). After a light meter (Omega/HHLM1337, Biel/Bienne, Switzerland) was used to measure the minimum lux of 14.48k lux in the photostability chamber, samples were exposed for 7 d (for a total of ∼2.6 × 10^6^ lux hours), and subsequently removed from the chamber. Light exposure of 7d was selected in order to exceed the minimum required 1.2 million lux hour exposure and also to minimize the degradation due to heat (produced by the lamps) by stretching the exposure over days instead of intense exposure over a short time frame. The contents of the samples were quantitatively transferred to a 250 mL volumetric flask using approximately 200 mL of diluent and sonicated until the contents were dissolved. The flask was diluted to volume with diluent and mixed. Per the Quinine Chemical Actinometry annex of ICH Q1B [12], the absorbance change of a 2% w/v quinine solutions was measured at 400 nm (Agilent Cary 60 UV using Starna Cells, Quartz Spectrophotometer Cell, 10 mm path length, Santa Clara, California, USA).

#### 2.3.3 Humidity degradation

Humidity degradation was focused only on the MPA powder and was not specifically evaluated for finished product because of the aqueous nature of the formulation. For MPA, 100 mg of powder was transferred into a tared glass vial. A saturated sodium carbonate solution (∼20 g in 15 mL of water) was prepared was added to a glass jar, capped, and allowed to sit at room temp for ∼30 min. Using a hygrometer (Vaisala /HM34, Vantaa, Finland), the humidity was measured inside the glass jar (∼92 % relative humidity) and exceeded the 75 % minimum level in ICH Q1A [11]. The sample vial was placed inside the vessel uncapped, but the large vessel was sealed leaving the sample exposed to the humidity within the large vessel for 14 d at RT. Afterwards, the contents of the vial were transferred to a 250 mL volumetric flask using approximately 200 mL of diluent and sonicate until the powder completely dissolved. The flask was diluted to volume with diluent and mixed.

#### 2.3.4 Heat degradation

For either MPA powder (100 mg) or resuspended finished product (1 mL), the required amount of sample was transferred into a tared vial. The vial was sealed and heated at approximately 80 °C for 14 d. The contents of the vial were transferred to a 250 mL volumetric flask using approximately 200 mL of diluent and sonicated until dissolved. The flask was diluted to volume with diluent and mixed.

#### 2.3.5 Acid degradation

For either MPA powder (100 mg) or resuspended finished product (1 mL), the required amount of sample was transferred into a 50 mL volumetric flask. The flask was filled with approximately 20 mL of diluent and 20 mL of 0.5 N hydrochloric acid solution. The mixture was sonicated until fully dissolved and diluted to volume with diluent. The flask remained at ambient conditions for 7 d (in ∼0.2 N HCl). Afterwards, the entire solution was transferred to a 250 mL volumetric flask, with the addition of 10 mL of 1.0 N sodium hydroxide. The flask was diluted to volume with diluent and mixed.

#### 2.3.6 Base degradation

For MPA API, 100 mg of powder was transferred into a 50 mL volumetric flask. The flask was filled with approximately 25 mL of diluent and 10 mL of 1.0 N sodium hydroxide solution. The solution was sonicated until the powder fully dissolved and subsequently diluted to volume with diluent. The solution was transferred to a plastic bottle (LDPE), capped, and remained at ambient conditions for 7 d. Afterwards, the entire solution was transferred to a 250 mL volumetric flask, with the addition of 20 mL of 0.5 N hydrochloric acid. The flask was diluted to volume with diluent and mixed.

For resuspended finished product, 1 mL of sample was transferred into a 50 mL plastic (polymethylpentene) volumetric flask. The flask was filled with approximately 25 mL of diluent and 5 mL of 1.0 N sodium hydroxide solution, diluted to volume with diluent, and mixed well. The flask remained at ambient conditions for approximately 4 h. Afterwards, the entire solution was transferred to a 250 mL volumetric flask with 10 mL of 0.5 N hydrochloric acid. The flask was diluted to volume with diluent and mixed.

#### 2.3.7 Oxidative degradation

For either MPA powder (100 mg) or resuspended finished product (1 mL), the required amount of sample was transferred into a 50 mL volumetric flask. The flask was filled with approximately 25 mL of diluent and 5 mL of 30 % hydrogen peroxide. The solution was sonicated until the sample was fully dissolved and the flask was diluted to volume with diluent and mixed. The flask was incubated at approximately 37 °C for 7 d. Afterwards, the flask was placed in the refrigerator (2−8 °C) until ready for analysis preparation. The entire solution was transferred to a 250 mL volumetric flask, diluted to volume with diluent and mixed.

### 2.4 Standard preparations

Working standards of MPA (0.4 mg/mL) were prepared by transferring 100 mg of MPA (USP reference standard) to a 250 mL volumetric flask with the addition of approximately 200 mL of diluent. The mixture was sonicated until completely dissolved, diluted to volume with diluent, and mixed well.

Reference solution A (1 mg/mL) was prepared by weighing 5 mg of MPA for system suitability (contains impurities A, B, C, D, E, G, I) into a 5 mL volumetric flask, diluting to volume with diluent and mixing well. Reference solution B (10 μg/mL) was prepared by transferring 250 μL of reference solution A to a 25 mL volumetric flask, diluting to volume with diluent, and mixing well. Reference solution C (1 μg/mL) was prepared by transferring 2.5 mL of reference solution B to a 25 mL volumetric flask, diluting to volume with diluent, and mixing well.

Impurity F reference solution A (2 mg/mL) was prepared by weighing 10 mg of MPA for peak identification into a 5 mL volumetric flask, diluting to volume with diluent, and mixing well. Impurity F reference solution B (20 μg/mL) was prepared by transferring 250 μL of impurity F reference solution A to a 25 mL volumetric flask, diluting to volume with diluent, and mixing well. Impurity F Reference Solution C (2 μg/mL) was prepared by transferring 2.5 mL of impurity F reference solution B to a 25 mL volumetric flask, diluting to volume with diluent, and mixing well.

### 2.5 HPLC-DAD-MS/MS analysis

#### 2.5.1 Instrument parameters

The chromatographic conditions used for the analysis were based on compendial methods for the API [8]. HPLC-DAD coupled with tandem mass spectrometry (MS/MS) was conducted with Agilent instrumentation using G1311A pump, G1329A autosampler, G1330A autosampler thermostat, G1316A column compartment, G1315B diode array detector, G6340 mass spectrometer modules (Agilent Technologies, Santa Clara, California, USA). Instrument software comprised Agilent ChemStation B.01.03 with 6300 Series Ion Trap LC/MS software Version 6.1. The following chromatographic conditions were used: 0.9 mL/min flow rate; Agilent column ZORBAX SB-C18 3.0 mm x 250 mm, 5 μm; 60 °C column temperature; 10 μL injection volume (MPA powder samples); 20 μL injection volume (finished product samples). The diode array detector (DAD) was set at 254 nm, where all spectra were stored from 190 nm to 400 nm. The mass spectrometer used the following parameters: capillary - 3500 V; Nebulizer 50.0 psi; Dry Gas 12.0 L/min; Dry Temp 350 °C; alternating polarity; Scan: 50–500 m/z; ESI Source.

#### 2.5.2 Mobile phase

Initial work used an isocratic mobile phase comprised of 65 % water, 23 % acetonitrile (ACN) and 12 % tetrahydrofuran (THF) based on compendial methods for the API [8]. Compendial methods for MPA injectable products that quantified specific impurities were not known when this work was initiated, but updates are available in the International Pharmacopoeia [10,13]. Due to challenges with impurity resolution using the isocratic mobile phase, a gradient mobile phase was used with the following timetable to improve the chromatographic resolution: 0 min, 76 % water, 16 % ACN, 8% THF; 30 min, 65 % water, 23 % ACN, 12 % THF; 50 min, 65 % water, 23 % ACN, 12 % THF; 50.1 min, 76 % water, 16 % ACN, 8% THF; 60 min, 76 % water, 16 % ACN, 8% THF.

### 2.6 Quantitative structure activity relationships (QSAR)

We followed the ICH M7(R1) guidelines recommending two *in silico* methods, one rule-based and another statistics-based, for the estimation of compound mutagenicity via prediction of the Ames assay result [14]. We used Toxtree 2.6.13 [15] as our rule-based method for the determination of the “Structural Alert for Genotoxic Carcinogenicity” endpoint [16] and ADMET Predictor® 9.0 [17] with the Toxicity module as our statistics-based method using quantitative structure-activity relationship (QSAR) techniques for determination of 10 Ames assay endpoints: TOX_MUT_97 + 1537, TOX_MUT_m97 + 1537, TOX_MUT_98, TOX_MUT_m98, TOX_MUT_100, TOX_MUT_m100, TOX_MUT_102+wp2, TOX_MUT_m102+wp2, TOX_MUT_1535, and TOX_MUT_m1535 (m denotes metabolic activation). We also calculated several acute toxicity endpoints using ADMET Predictor® models, including rat estrogen receptor toxicity (ER), rat androgen receptor toxicity (AR), rat allergenic respiratory sensitization, human ether-a-go-go related gene (hERG) inhibition, phospholipidosis, and hepatoxicity through 5 liver enzymes: alkaline phosphatase (Ser_AlkPhos), gamma-glutamyltransferase (Ser_GGT), lactate dehydrogenase (Ser_LDH), aspartate aminotransferase (Ser_AST), and alanine aminotransferase (Ser_ALT), as well as a non-mutagenic genotoxicity endpoint for chromosomal aberrations. ADMET Predictor® model performance is shown in Supplemental Table 1 [17]. All ADMET Predictor® values presented herein were from compounds that fell within the applicability domain of the specific endpoint model.

## 3 Results and discussion

**Fig. 2:**
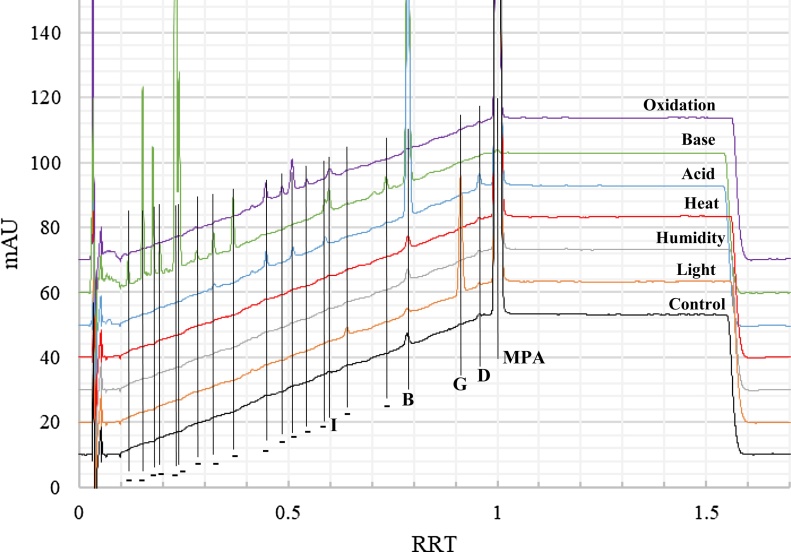
Overlaid chromatograms for API exposed to various forced degradation conditions: Control (maintained at ambient); light (2.6 × 10^6^ lux hours); humidity (14d at 92 %RH); heat (14d at 80 °C); acid (7d in 0.2 N HCl); base (7d in 0.2 N NaOH); oxidation (7d in 3% H_2_O_2_ at 37 °C). Total run times were 60 min, corresponding to an RRT of approximately 1.7. Peak labels indicate known impurities. “- “indicates an unknown degradation product for the API.

**Fig. 3:**
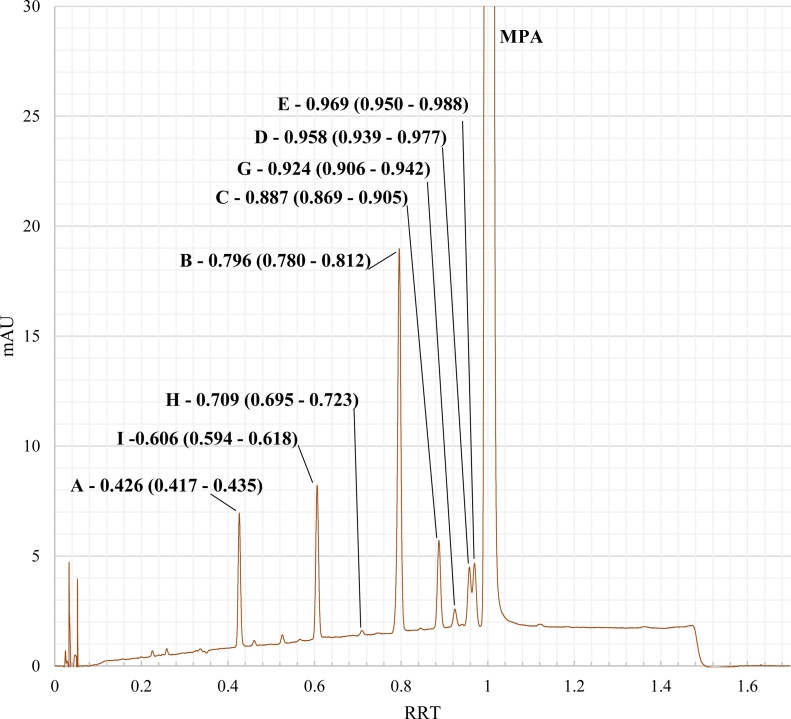
Example chromatogram for Reference Solution A with assignments for respective medroxyprogesterone impurities [8,10,18], including relative retention times and +/- 2% RRT range observed for implemented chromatographic conditions.

Forced degradation results for MPA active ingredient are provided in Table 1 with example chromatograms in Fig. 2, where the peak identifications for impurities A–E and G–I are based on the injection of reference solution A (Fig. 3). Relative to the control sample, base exposure generated the most impurities and degradation products, followed in decreasing order by acid, oxidative, and light exposure. Exposure to humidity and heat generated no additional impurities or degradation products that were readily observable relative to the control. Impurities A, C, E, F, and H were not observed in any of the MPA active ingredient samples, including the control. The chromatographic conditions used were based on those employed for known impurities A–E and G–I [8], where impurity F has separate methods available for characterization [[7], [8], [9], [10]]. Levels for impurity I increased with base and oxidative exposure, while impurity B increased with both acid and base exposure. Impurity levels for D and G increased when subjected to acid and light exposure, respectively.

**Table 1:** Results summary (resolution; % area count) for different forced degradation conditions for MPA for observed relative retention times (RRT).^a^, ^b^.

Peak ID^c^	RRT^d^	Control (Ambient)	Light	Humidity	Heat	Acid	Base	Oxidative
–	0.12	NP	NP	NP	NP	NP	7.4; 1.1 %	NP
–	0.15	NP	NP	NP	NP	NP	6.0; 7.2 %	NP
–	0.18	NP	NP	NP	NP	NP	3.8; 5.0 %	NP
–	0.19	NP	NP	NP	NP	NP	2.7; 1.2 %	NP
–	0.23	NP	NP	NP	NP	NP	5.2; 39.1 %	NP
–	0.24	NP	NP	NP	NP	NP	1.1; 9.0 %	NP
–	0.28	NP	NP	NP	NP	NP	4.5; 0.6 %	NP
–	0.32	NP	NP	NP	NP	n/a; 0.3 %	4.3; 1.2 %	NP
–	0.37	NP	NP	NP	NP	NP	5.0; 2.9 %	NP
A – 0.426 (0.417−0.435)	0.43	NP	NP	NP	NP	NP	NP	NP
–	0.45	NP	NP	NP	NP	3.6; 0.7 %	NP	18.6; 2.3 %
–	0.49	NP	NP	NP	NP	NP	NP	3.4; 0.9 %
–	0.51	NP	NP	NP	NP	5.3; 0.4 %	NP	2.0; 3.9 %
–	0.54	NP	NP	NP	NP	NP	NP	2.7; 0.6 %
–	0.59	NP	NP	NP	NP	5.9; 0.5 %	20.9; 0.9 %	NP
I – 0.606 (0.594−0.618)	0.60	NP	NP	NP	NP	NP	0.7; 1.7 %	3.4; 2.0 %
–	0.64	NP	n/a; 0.3 %	NP	NP	NP	NP	NP
H – 0.709 (0.695 – 0.723)	0.71	NP	NP	NP	NP	NP	NP	NP
–	0.74	NP	NP	NP	NP	NP	10.2; 1.2 %	NP
B – 0.796 (0.780 – 0.812)	0.79	n/a; 0.5 %	6.7; 0.3 %	n/a; 0.5 %	n/a; 0.6 %	14.7; 10.7 %	3.9; 28.8 %	NP
C – 0.887 (0.869 – 0.905)	0.89	NP	NP	NP	NP	NP	NP	NP
G – 0.924 (0.906−0.942)	0.91	NP	5.9; 4.4 %	NP	NP	NP	NP	NP
D – 0.958 (0.939 – 0.977)	0.96	NP	NP	NP	NP	12.1; 0.6 %	NP	NP
E – 0.969 (0.950 – 0.988)	0.97	NP	NP	NP	NP	NP	NP	NP
MPA	1.00	13.5; 99.5 %	5.9; 95.0 %	13.7; 99.5 %	13.5; 99.4 %	2.9; 87.0 %	n/a; 0.0 %	22.2; 90.3 %

NP - no readily integrable peak.

a – Peak purity (PP) data was collected for peaks with large enough area counts, where the average result was 987.7 with a relative standard deviation of 1.71 %.

b – Non-degraded (maintained at ambient); light (2.6 × 10^6^ lux hours); humidity (14d at 92 %RH); heat (14d at 80 °C); acid (7d in 0.2 N HCl); base (7d in 0.2 N NaOH); oxidative (7d in 3% H_2_O_2_ at 37 °C).

c – Proposed assignments based on observed relative retention times (RRT) for impurities in Reference solution A injections, including +/- 2% RRT range observed (see Fig. 3). ‘- ‘indicates an unknown degradation product for the API.

d – Average relative retention times observed for different forced degradation sample injections.

**Fig. 4:**
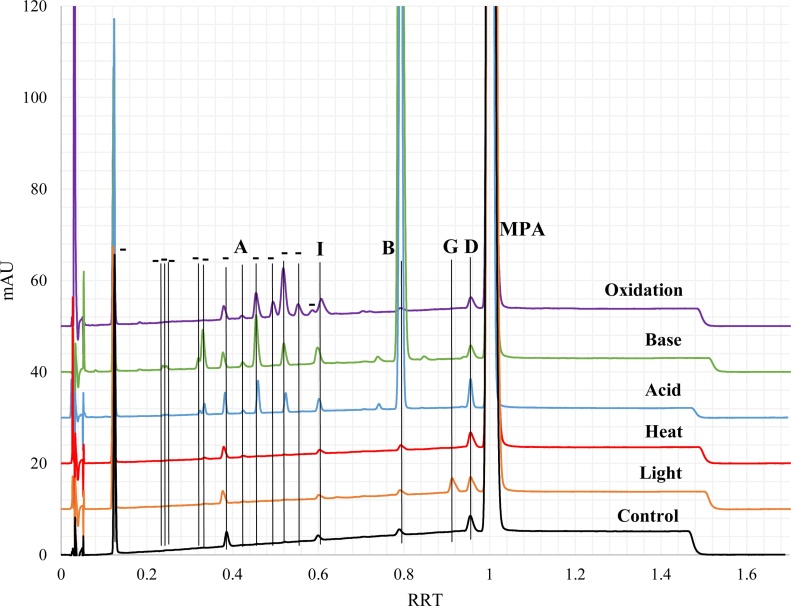
Overlaid chromatograms for sample 183 exposed to various forced degradation conditions: Control (maintained at ambient); light (2.6 × 10^6^ lux hours); heat (14d at 80 °C); acid (7d in 0.2 N HCl); base (4 h in 0.1 N NaOH); oxidation (7d in 3% H_2_O_2_ at 37 °C). Peak labels indicate known impurities. “- “indicates either degradation product or excipient, where further identifications are proposed in Table 3 in conjunction with MS observations.

Results for injectable products exposed to various forced degradation conditions are shown in Table 2 with example chromatograms provided in Fig. 4 and Supplemental Figs. 1–3 for samples 183, 123, 789, and 890, respectively. Due to the essentially complete degradation observed for the MPA active ingredient with basic conditions, the exposure time and concentration of NaOH were reduced for injectable product samples. Even with this change, base exposure yielded the most impurities and degradation products for injectable products, as observed for the MPA active ingredient. Similar to the results for the MPA active ingredient, acidic and oxidative conditions yielded the second highest number of impurities and degradation products, followed by light exposure. Heat exposure provided a comparable number of impurities / degradation products relative to the control samples. Regardless of the components observed, the general amounts observed (measured in % area count) for each specific impurity / degradation product were at comparable levels across all samples (123, 183, 789, and 890). There were several impurities not readily observed in some samples but were observed at minimal levels (typically ∼0.1 %) in the other samples.

**Table 2:** Results summary (sample - average resolution; average % area count) for different forced degradation conditions for finished product samples 123, 183, 789, and 890.^a^, ^b^.

Peak ID^c^	RRT^d^	Control (Ambient)	Light	Heat	Acid	Base	Oxidative
–	0.12	123– 12.0; 2.9 % 183– 12.4; 2.5 % 789−12.1; 2.5 % 890−12.1; 2.2 %	123– 10.9; 2.8 % 183– 10.6; 2.3 % 789−10.7; 2.4 % 890−10.7; 2.0 %	123– 11.0; 2.6 % 183– 11.0; 1.9 % 789−11.0; 2.1 % 890−10.9; 1.8 %	123−14.3; 2.9 % 183−11.7; 2.5 % 789−14.2; 2.7 % 890−14.2; 2.2 %	123−15.0; 2.8 % 183−13.6; 2.4 % 789−13.3; 2.6 % 890−14.2; 2.1 %	123−8.0; 3.3 % 183– 9.5; 3.0 % 789−10.9; 3.9 % 890−11.1; 3.1 %
–	0.15	NP	NP	NP	NP	NP	NP
–	0.18	NP	NP	NP	NP	NP	NP
–	0.19	NP	NP	NP	NP	NP	NP
–	0.23	NP	NP	NP	NP	123−15.5; 0.1 %^e^ 183- NP 789- NP 890- NP	NP
–	0.24	NP	NP	NP	NP	123−4.8; 0.1 % 183- NP 789- NP 890−15.5; 0.04 %^e^	NP
	0.25	NP	NP	NP	NP	123−0.7; 0.1 %^e^ 183- NP 789- NP 890- NP	NP
–	0.28	NP	NP	NP	NP	NP	NP
–	0.32	NP	NP	NP	NP	123−7.1; 0.1 % 183−21.3; 0.1 % 789−21.8; 0.1 %^e^ 890−18.2; 0.1 %	NP
–	0.33−0.34	NP	NP	NP	123−29.2; 0.1 %^e^ 183– 29.1; 0.1 % 789−29.6; 0.1 %^e^ 890−29.3; 0.1 %^e^	123−0.9; 0.6 % 183−0.8; 0.6 % 789−0.8; 0.6 % 890−1.0; 0.5 %	NP
–	0.37	NP	NP	NP	NP	NP	NP
–	0.38−0.39	123– 25.9; 0.3 % 183– 26.5; 0.2 % 789−26.1; 0.2 % 890−25.8; 0.2 %	123– 22.8; 0.2 % 183−22.2; 0.2 % 789−22.2; 0.2 % 890−23.0; 0.2 %	123– 24.4; 0.2 % 183– 23.9; 0.2 % 789−24.1; 0.2 % 890−24.4; 0.2 %	123−15.3; 0.3 % 183– 5.2; 0.2 % 789−15.1; 0.2 % 890−5.6; 0.2 %	123−4.5; 0.3 % 183−3.5; 0.2 % 789−3.6; 0.2 % 890−4.9; 0.2 %	123−22.1; 0.4 % 183– 22.3; 0.4 % 789−22.4; 0.4 % 890−21.9; 0.3 %
A – 0.426 (0.417−0.435)	0.42−0.43	NP	NP	NP	123−4.0; 0.1 % 183 – NP 789- NP 890−3.9; 0.1 %^e^	123−3.7; 0.1 % 183 – NP 789 – NP 890−3.8; 0.1 %	NP
–	0.45−0.46	NP	NP	NP	123−3.4; 0.2 % 183– 7.7; 0.4 % 789−7.5; 0.4 % 890−6.6; 0.4 %	123– 2.7; 0.8 % 183– 5.7; 0.8 % 789−5.7; 0.8 % 890−3.0; 0.8 %	123−4.5; 1.2 % 183– 4.6; 0.7 % 789−4.6; 0.8 % 890−4.5; 0.9 %
–	0.49	NP	NP	NP	NP	NP	123−2.4; 0.6 %^e^ 183– 2.4; 0.4 % 789−2.3; 0.5 % 890−2.3; 0.6 %^e^
–	0.51	NP	NP	NP	NP	NP	NP
–	0.52	NP	NP	NP	123−5.9; 0.2 %^e^ 183– 6.0; 0.2 % 789−5.9; 0.2 %^e^ 890−5.9; 0.2 %	123−5.2; 0.4 %^e^ 183– 4.3; 0.4 % 789−4.4; 0.4 % 890−5.5; 0.4 %	123−1.4; 2.6 %^e^ 183– 1.4; 1.6 % 789−1.3; 1.8 % 890−1.3; 2.1 %
–	0.54	NP	NP	NP	NP	NP	NP
–	0.55−0.56	NP	NP	NP	NP	NP	123−1.8; 0.5 % 183– 1.8; 0.4 % 789−1.8; 0.4 % 890−1.8; 0.5 %
–	0.59	NP	NP	NP	NP	NP	NP
I – 0.606 (0.594−0.618)	0.60	123- NP 183- NP 789−14.6; 0.1 % 890- NP	NP	NP	123−6.4; 0.1 % 183– 6.0; 0.2 % 789−10.9; 0.2 % 890−6.4; 0.1 %	123−5.3; 0.4 % 183– 4.4; 0.3 % 789−4.4; 0.4 % 890−5.8; 0.3 %	123−1.7;0.9 % 183– 2.6; 0.5 % 789−2.6; 0.6 % 890−2.6; 0.7 %
–	0.64	NP	NP	NP	NP	NP	NP
H – 0.709 (0.695−0.723)	0.71	NP	NP	NP	NP	NP	NP
–	0.74	NP	NP	NP	123 - NP 183– 10.3; 0.1 % 789 - NP 890 - NP	NP	NP
B – 0.796 (0.780 – 0.812)	0.79−0.81	NP	NP	NP	123−14.6; 6.5 % 183– 4.0; 11.2 % 789−14.4; 7.0 % 890−11.5; 10.8 %	123−12.3; 25.1 % 183– 9.7; 25.3 % 789−9.6; 25.4 % 890−13.7; 24.5 %	NP
–	0.86	NP	NP	NP	NP	NP	NP
C – 0.887 (0.869 – 0.905)	0.89	NP	NP	NP	NP	NP	NP
G – 0.924 (0.906−0.942)	0.91−0.93	NP	123– 28.9; 0.6 % 183−28.1; 0.4 % 789−28.7; 0.3 % 890−29.6; 0.3 %	NP	NP	NP	NP
D – 0.958 (0.939 – 0.977)	0.96	123 - NP 183 – 34.4; 0.4 % 789- NP 890−33.8; 0.2 %^e^	123 - NP 183– 1.9; 0.4 % 789- NP 890−2.0; 0.3 %^e^	123 - NP 183– 32.4; 0.4 % 789- NP 890−34.8; 0.2 %	123−11.7; 0.3 % 183– 11.8; 0.5 % 789−11.6; 0.5 % 890−11.7; 0.4 %	123 - NP 183– 8.7; 0.2 % 789- NP 890−11.3; 0.2 %	123 - NP 183– 15.8; 0.4 % 789- NP 890- NP
E – 0.969 (0.950 – 0.988)	0.97	NP	NP	NP	NP	NP	NP
MPA	1.00	123- 33.6; 96.9 % 183−2.1; 96.9 % 789−20.0; 97.2 % 890−12.3; 97.5 %	123– 3.5; 96.4 % 183– 1.7; 96.7 % 789−3.4; 97.1 % 890−3.0; 97.4 %	123−32.5; 97.2 % 183−1.9; 97.6 % 789−31.7; 97.7 % 890−7.3; 97.9 %	123−2.8; 89.3 % 183– 2.9; 84.7 % 789−2.8; 89.0 % 890−2.8; 85.5 %	123−12.4; 69.4 % 183– 2.1; 69.6 % 789−10.3; 69.6 % 890−2.8; 70.8 %	123−15.8; 90.6 % 183– 1.9; 92.6 % 789−16.0; 91.6 % 890−16.6; 91.8 %

NP – no readily integrable peak.

a – Peak purity (PP) data was collected for peaks with large enough area counts, where the average / %RSD for the different samples were obtained as indicated (123–999.9 / 0.01 %; 183–999.9 / 0.01 %; 789–998.2 / 1.25 %; 890–999.8 / 0.1 %).

b – Non-degraded (maintained at ambient); light (2.6 × 10^6^ lux hours); heat (14d at 80 °C); acid (7d in 0.2 N HCl); base (4 h in 0.1 N NaOH); oxidative (7d in 3% H_2_O_2_ at 37 °C).

c – Proposed assignments based on observed relative retention times (RRT) for impurities in Reference solution A injections, including +/- 2% RRT range observed (see Fig. 3). ‘- ‘indicates either degradation product or excipient, where further identifications are proposed in Table 3 in conjunction with MS observations.

d – Average relative retention times observed for different forced degradation sample injections.

e – Peak was not present in all injects across the various samples.

Similar to the results for the MPA active ingredient, impurities C, E, F, and H were not observed in any of the MPA finished product samples (including the control). However, impurity A was found at low levels (∼0.1 %) under acidic and basic conditions for samples 123 and 890, but not for samples 183 and 789. Levels for impurity I increased more prominently with base and oxidative exposure (similar to the MPA active ingredient), with a minor increase under acidic conditions. As with MPA active ingredient, impurity B increased with both acid and base exposure, while impurities D and G increased with acid and light exposure, respectively.

Coupled with the peak assignments previously made based on the reference solution A injections, additional peak identifications are indicated in Table 3, where available molecular weight data (both reference molecular weights and from tandem MS/MS data) are provided for the different peaks observed in the injectable product samples. Peaks at RRT of ∼0.12 and ∼0.38 have been assigned to methylparaben and propylparaben, respectively, based on molecular weight data and that these compounds are typically used as preservatives in these formulations [21,22]. In addition to parabens, other excipients for various sources of MPA injectables (150 mg/mL) are polyethylene glycol 3350, polysorbate 80, sodium chloride, sodium hydroxide/hydrochloric acid (final pH adjustment), and water [21], where macrogol 4000 can also be utilized [22].

**Table 3:** Proposed peak identifications for analytes observed in finished product chromatograms as determined from assignments using reference solution A and available molecular weight data.

Peak ID^a^	RRT^b^	Reference Mol. Wt. (g/mol)^e^	Detected m/z values for protonated molecular and adduct ions
Methylparaben	0.12	152.2	[M-H]^+^: 152, [M + Na-2 H]^+^: 173
Unknown 1^c^	0.23	–	f
Unknown 2^c^	0.24	–	[M+H]^+^: 326
Unknown 3^c^	0.25	–	f
Proposed Structures-Group 1^d^	0.32	376.5	[M+H]^+^: 378, [M + Na]^+^: 400
	0.33−0.34		
Propylparaben	0.38−0.39	180.2	[M-H]^+^: 179
A – 0.426 (0.417−0.435)	0.42−0.43	402.5	[M + Na]^+^ 426
Proposed Structures-Group 2^d^	0.45−0.46	418.5	[M+H]^+^: 419, [M + Na]^+^: 441 [M + K]^+^: 457
Proposed Structures-Group 3^d^	0.49	420.5	[M+H]^+^: 421
Proposed Structures-Group 2^d^	0.52	418.5	[M+H]^+^: 419, [M + Na]^+^: 441 [M + K]^+^: 457
Proposed Structures-Group 3^d^	0.55−0.56	420.5	[M+H]^+^: 421, [M + Na]^+^: 443 [M + K]^+^: 459
I – 0.606 (0.594−0.618)	0.60	344.5	[M + ACN+H]^+^: 387, [M + ACN + Na]^+^: 409
H – 0.709 (0.695−0.723)	0.71	372.5	f
Unknown 4	0.74	–	f
B – 0.796 (0.780 – 0.812)	0.79−0.81	344.5	[M+H]^+^: 345, [M + Na]^+^: 367
C – 0.887 (0.869 – 0.905)	0.89	386.5	f
G – 0.924 (0.906−0.942)	0.91−0.93	384.5	[M + Na]^+^: 407
D – 0.958 (0.939 – 0.977)	0.96	386.5	[M+H]^+^: 387, [M + Na]^+^: 409, [M + K]^+^: 425
E – 0.969 (0.950 – 0.988)	0.97	384.5	f
MPA	1.00	386.5	[M+H]^+^: 387

a – Proposed assignments for impurities A–E and G–I based on observed relative retention times (RRT) for impurities in Reference solution A injections, including +/- 2% RRT range observed (see Fig. 3).

b – Relative retention times observed for different forced degradation sample injections.

c – Unknowns 1–3 may be attributable to polysorbate 80 [19].

d – proposed structures as observed in Figure 5 (with appropriate reference).

e – Molecular Weight from literature sources [20]. Additional information available at https://capri.ctiexchange.org.

f – no clearly discernable dominant molecular weight observed.

Unknowns are present in the base degradation chromatogram for MPA API at large quantities at RRT 0.23 and 0.24 (39.1 % and 9.0 %, respectively), but in much smaller quantities for MPA finished products at RRT 0.23−0.25, and only for certain product runs. We note that the base degradation exposure procedure for the finished products was modified from that used on the MPA API, causing a difference in the observed degradation products and their relative intensities. Certain ion peaks present in the finished product spectra for these unknowns suggest that the unknown is polysorbate 80 or a degradant thereof [19], but the presence of these peaks in the MPA API chromatograms precludes that possibility. Currently we do not have enough information to identify these unknowns.

Additionally, other peaks outlined in Table 3 (with supporting mass spectra shown in Supplemental Fig. 4) have potential structures that are tentatively proposed in Fig. 5 in three different groups through various hydrolysis or oxidative processes that are combinations of hydroxyl substitutions or deacetylations (some correlating with those proposed in metabolism studies [23]) with molecular weights that align with observed *m/z* values. For Group 1, 2,6-dihydroxymedroxyprogesterone and/or its isomers may be generated in both acid and base degradations at RRT 0.32 to 0.33. Mass data ([M+H]^+^, 378 *m/z*) suggested that these peaks are the result of deacetylation of MPA with possible addition of two hydroxyl groups. For Group 2, 2,6-dihydroxymedroxyprogesterone acetate and/or its isomers may be found in both the acid and base degradation samples at RRT 0.45 and 0.52. The mass spectrum shows a parent ion of 419 *m/z* [M+H]^+^, with 441 *m/z* [M + Na]^+^, and 457 *m/z* [M + K]^+^. This may indicate an addition of two hydroxyl groups to medroxyprogesterone (MPA - 2H + 2OH) and could be a result of acid-catalyzed enol formation or base-catalyzed enolate formation, followed by hydrolysis. The likely positions for this substitution are at C2, C6 (due to conjugation with the C3 carbonyl), and C21 due to their relatively low pKa values. Within Group 3, various diastereomers of 4,5-dihydroxymedroxyprogesterone acetate may result from oxidative degradation of samples in the aqueous conditions at RRT 0.49 and 0.56. The mass spectrum shows that the parent ions for both peaks are 421 *m/z* [M+H]^+^, 443 *m/z* [M + Na]^+^ and 459 *m/z* [M + K]^+^. The parent mass suggests MPA with an addition of two hydroxyl groups (MPA + 2OH). These diastereomeric structures would be the result of the oxidation of the double bond in the A ring of MPA. The separation observed between the two peaks in the HPLC chromatogram is the result of the differing chemical properties of the diastereomers. Although these structures may be reasonable proposals, no further confirmation has been obtained and they were not included in subsequent QSAR analysis. Furthermore, these proposed structures may have very limited stability and other types than the ones proposed above could also be possible.

**Fig. 5:**
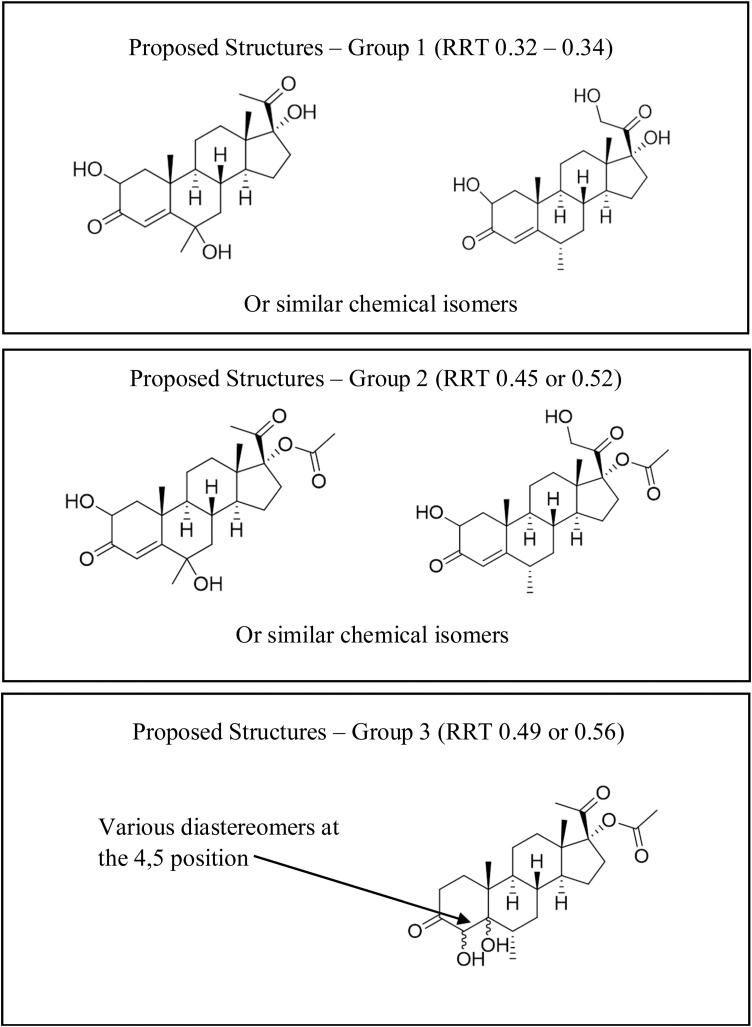
Proposed structures for various impurities observed in chromatograms (see Table 3).

In comparing the potential impact of excipients, no major differences were observed in the prominent types of impurities and degradation products found in samples of API and finished product for light, thermal and oxidative degradation conditions (in comparison to respective control samples). For acid exposure, impurities B and D were the most prominent impurities generated for either API or finished product, including the presence of degradation products at RRT of 0.32−0.34, 0.45. However, excipients may have had an impact (exact reason unknown) on shifting the degradation products with acid exposure because degradations at RRT of 0.51 and 0.59 were present in API, but absent in finished product, with degradation products observed in finished product at RRT of 0.52 and 0.6 (impurity I). For base conditions, it is difficult to make a direct comparison for the impact of excipients because the concentration and time were lessened for finished product due to the essentially complete degradation of MPA for the API only samples. Regardless, impurity B was still the same primary known impurity observed for either case. Although it is difficult to draw conclusions for many of the peaks under base exposure because of the complete degradation of API and the presence of the paraben peaks, a major difference in finished product samples under base exposure is the presence of the peaks proposed for Group 2, with their absence in the API samples. The exact reason for this difference is not confirmed, but it may be possible that they are generated in the API sample but further deacetylated to the Group 1 compounds in the stronger base conditions and not actually due to the excipients.

ADMET Predictor® 9.0 predicted values for the Ames mutagenicity of MPA and its known impurities are listed in Supplemental Table 2. Values are given for five different *Salmonella typhimurium* strains, both with and without metabolic activation. All compounds were predicted to be negative for all strains. Toxtree structural alerts for these compounds are given in Supplemental Table 3. MPA and all impurities except 4,5-dihydromedroxyprogesterone acetate (Impurity F) were flagged for a genotoxic carcinogenicity (mutagenicity) structural alert due to the presence of an α,β-unsaturated carbonyl, corresponding to the carbonyl at C3 and double bond at C4-C5. No structural alerts were found for non-genotoxic carcinogenicity.

ADMET Predictor® values for non-genotoxic endpoints are given in Table 4. All compounds were predicted to not bind significantly to the rat estrogen receptor (denoted as being “Nontoxic”), while all compounds except Impurity C were predicted to bind significantly to the rat androgen receptor (denoted as “Toxic”). All compounds were predicted to be rat allergenic respiratory “nonsensitizers”, not inhibitors of hERG, “nontoxic” for inducing phospholipidosis, and “toxic” (predicted to result in a positive assay) for chromosomal aberrations. For hepatotoxicity endpoints, MPA and all impurities except B, C, and I were predicted to cause elevated serum alkaline phosphatase, all except impurity F were predicted to cause elevated LDH, and all were predicted to cause elevated ALT. Impurity G was the only compound predicted to cause elevated GGT, and impurities B, G, and I were the only compounds predicted to have elevated AST.

**Table 4:** ADMET Predictor® results for MPA and its impurities for several other toxicological models: rat estrogen receptor toxicity (ER), rat androgen receptor toxicity (AR), allergenic respiratory sensitization in rat, human ether-a-go-go-related gene (hERG) inhibition, phospholipidosis, and hepatotoxicity through five serum liver enzyme models: alkaline phosphatase (Ser_AlkPhos), gamma-glutamyltransferase (Ser_GGT), lactate dehydrogenase (Ser_LDH), aspartate aminotransferase (Ser_AST), and alanine aminotransferase (Ser_ALT). Values in parenthesis denote ADMET Predictor™ confidence in prediction.

ID	Chromosomal Aberrations	ER	AR	Respiratory Sensitization	hERG Inhibition	Phospholipidosis	Ser_AlkPhos	Ser_GGT	Ser_LDH	Ser_AST	Ser_ALT
MPA	Toxic (79 %)	Nontoxic (83 %)	Toxic (68 %)	Nonsensit. (92 %)	No (96 %)	Nontoxic (99 %)	Elevated (54 %)	Normal (86 %)	Elevated (78 %)	Normal (98 %)	Elevated (94 %)
A	Toxic (73 %)	Nontoxic (83 %)	Toxic (63 %)	Nonsensit. (94 %)	No (96 %)	Nontoxic (99 %)	Elevated (78 %)	Normal (80 %)	Elevated (47 %)	Normal (57 %)	Elevated (94 %)
B	Toxic (82 %)	Nontoxic (80 %)	Toxic (93 %)	Nonsensit. (99 %)	No (77 %)	Nontoxic (99 %)	Normal (70 %)	Normal (97 %)	Elevated (78 %)	Elevated (42 %)	Elevated (72 %)
C	Toxic (82 %)	Nontoxic (77 %)	Nontoxic (59 %)	Nonsensit. (92 %)	No (96 %)	Nontoxic (99 %)	Normal (55 %)	Normal (97 %)	Elevated (78 %)	Normal (91 %)	Elevated (94 %)
D	Toxic (79 %)	Nontoxic (83 %)	Toxic (68 %)	Nonsensit. (92 %)	No (96 %)	Nontoxic (99 %)	Elevated (54 %)	Normal (86 %)	Elevated (78 %)	Normal (98 %)	Elevated (94 %)
E	Toxic (97 %)	Nontoxic (80 %)	Toxic (93 %)	Nonsensit. (98 %)	No (96 %)	Nontoxic (99 %)	Elevated (57 %)	Normal (65 %)	Elevated (78 %)	Normal (78 %)	Elevated (94 %)
F	Toxic (60 %)	Nontoxic (77 %)	Toxic (66 %)	Nonsensit. (92 %)	No (96 %)	Nontoxic (99 %)	Elevated (59 %)	Normal (97 %)	Normal (77 %)	Normal (98 %)	Elevated (49 %)
G	Toxic (91 %)	Nontoxic (83 %)	Toxic (68 %)	Nonsensit. (92 %)	No (96 %)	Nontoxic (99 %)	Elevated (59 %)	Elevated (72 %)	Elevated (78 %)	Elevated (60 %)	Elevated (94 %)
H	Toxic (91 %)	Nontoxic (77 %)	Toxic (63 %)	Nonsensit. (98 %)	No (96 %)	Nontoxic (99 %)	Elevated (50 %)	Normal (90 %)	Elevated (78 %)	Normal (98 %)	Elevated (94 %)
I	Toxic (82 %)	Nontoxic (80 %)	Toxic (51 %)	Nonsensit. (99 %)	No (77 %)	Nontoxic (99 %)	Normal (68 %)	Normal (90 %)	Elevated (78 %)	Elevated (53 %)	Elevated (72 %)

For MPA injectable products, the formulation is commonly in a clear glass vial sealed with a cap (primary packaging) that allows subsequent puncture with a syringe needle for injection at time of use. Furthermore, the vial(s) are typically placed inside a small box (secondary packaging), often with a package insert depending on the client / market needs. The vial contains a sterile aqueous suspension that requires re-suspension before injection. Before resuspension, it is common to observe a white layer of solids settled at the bottom of the vial, while the remainder of the upper layer is a clear liquid.

With the primary and secondary packaging, the various force degradation categories employed in this study could be considered to have different levels of risk during actual product storage and use. With secondary packaging, the vials have minimal risk to light exposure, assuming the vial remains in the box until time of administration. Risk to oxidation could be considered minimal due to the sealed vial. Generally, the primary oxygen source will result from any level present during the time of manufacture and could be further minimalized if gaseous purges are implemented during production. These products are typically manufactured to yield a pH just under 7.0, where the pH specification range is 3.0–7.0 [10]. Therefore, this product can be expected to exist under mildly acidic conditions (pH of 3.0–7.0) throughout the shelf-life and maintain compliance with compendial criteria. With properly manufactured injectable products (formulated and sealed), the risk of exposure to strong acidic/basic conditions is minimal. MPA injectable products would be at no greater risk of humidity exposure in tropical environments because the product is an aqueous suspension and is constantly exposed to water regardless of the environmental humidity. The most likely condition that these products will be subjected to during transport, storage, and use within tropical environments is thermal exposure. Although the objective of this forced degradation study is to understand the range of compounds that could be created rather than being a traditional stability study (to understand what compounds are actually generated in expected environmental conditions over a longer time period), the injectable products evaluated appeared less prone to thermal degradation relative to acid/base, oxidation, and light exposures. Moreover, thermal forced degradation conditions essentially resulted in no change to the impurity or degradation product levels relative to the control for all of the products. If impurities B, D, G, or I are observed in products using the Ph. Int. monograph [10], it may be an indicator of exposure to acid/base, light, or oxidation. Besides observed impurity / degradation product levels, it is important to note that other parameters for this type of product may change with environmental exposures and should also be monitored, such as volume of injection (resuspendability) and pH [10].

MPA has been approved for use in drug products for decades and some information regarding its toxicity is publicly available. However, toxicity data on pharmaceutical impurities / degradation products are largely kept proprietary [24] and public information is practically non-existent. Therefore, we have performed QSAR analysis on MPA and its impurities to estimate the level of risk that exposure to these compounds may generate.

Potential genotoxicity and carcinogenicity are the largest concerns in assessing the toxicology of a compound [16]. That none of the pharmacopoeia monograph impurity limits for MPA API nor injectable are set according to the 1.5 μg/day threshold of therapeutic concern for carcinogenicity as described in ICH M7(R1) suggests that all compounds were found, or at least not known, to be non-mutagenic during regulatory approval processes for MPA drug products. Toxtree flagged MPA and all impurities except impurity F as having a genotoxic carcinogenicity structural alert for an α,β-unsaturated carbonyl (Supplemental Table 3), corresponding to the carbonyl at C3 and double bond between C4 and C5. ADMET Predictor®, however, predicts negative Ames mutagenicity for MPA and all its impurities for every strand of *Salmonella* tested, with and without metabolic activation (Supplemental Table 2). MPA itself has been experimentally tested and found to be non-mutagenic in the Ames assay [25]. As MPA shares its structural alert with most of its impurities but is non-mutagenic, these impurities would be categorized as Class 4 and treated as non-mutagenic, per ICH M7(R1). Therefore, MPA impurities would be subject to normal identification and qualification limits as described in ICH Q3A [26] and Q3B [27] and pose little risk for genotoxic mutagenicity and carcinogenicity.

ADMET Predictor® results for non-mutagenic toxicity endpoints are shown in Table 4. The result for the non-mutagenic genotoxicity endpoint chromosomal aberrations showing MPA as toxic (predicted to be positive in the assay) is consistent with reports in the literature. Some animal studies have shown that MPA, as well as other natural and synthetic progestogens including progesterone, can produce chromosomal aberrations, but how this translates to humans is unclear [28], and ultimately these compounds are considered relatively safe. Therefore, we consider the risk for chromosomal aberrations posed by MPA impurities to be similar to MPA itself.

Two other potential toxicity concerns are highlighted by the ADMET Predictor® results in Table 4. First, all compounds except impurity C were predicted to bind significantly to the rat androgen receptor (AR). MPA binding to the AR is known; it is generally considered a weak AR agonist [29]. While its androgenic behavior is undesirable, it is thought of as less of a toxicological risk and more as a source of side effects such as hirsutism and acne. The second concern is the prediction that MPA and its impurities cause elevation of certain serum liver enzyme concentrations. Our results predict elevated alkaline phosphatase, LDH, and ALT for MPA. The FDA-approved label for Depo-Provera® acknowledges the possibility of elevated serum liver enzymes and recommends discontinuation of the product until levels return to normal if “disturbances of liver function develop” [21]. As most MPA impurities are predicted to cause some combination of elevated liver enzymes (primarily alkaline phosphatase, LDH and ALT, like MPA), the hepatotoxicity risk for these compounds is likely similar to that of MPA. Impurity G, megestrol acetate (MGA), was predicted to cause elevated levels of all five liver enzymes, but as this compound is itself an approved drug, the risk it poses as an impurity is low, even at amounts observed in our forced degradation studies (4.4 % under light degradation of MPA API, up to 0.6 % under light degradation of MPA).

Overall, our predicted toxicity results suggest that MPA impurities are no more toxic than MPA itself. Although there is some variation between limits set for MPA impurities by different pharmacopoeias, the 1% threshold for all impurities except Impurity F (0.5 % limit) set by the BP and Ph. Int. MPA injectables monographs, as well as the USP MPA API monograph, appears high enough given the apparent low toxicity risk for these compounds. On the other hand, the BP, Ph. Int., and EP limits for MPA API are set tightly for Impurities C, E, G, and I at 0.2 %; this would fall at or under the qualification limit for impurities in drug products, depending on the dosage of the product, meaning that safety data would not even be required at these levels. There appears to be no safety reasons why these impurities cannot be set higher. In such cases it is likely that these impurity limits are set entirely or in part by the analytical and process capabilities of the API manufacturer [30], which is a justification explicitly allowed for in the ICH guidelines. This is supported by the fact that MPA shares two impurities with MGA, a related progestin and approved drug, with each compound having different BP impurity limits in the two different API monographs. 6-Methylenehydroxyprogesterone acetate (6-methylidene-3,20-dioxopregn-4-en-17-yl acetate) has a limit of 0.2 % in the BP MPA API monograph as Impurity E and 0.3 % in the BP MGA API monograph as Impurity D, whereas 6-epimedroxyprogesterone acetate (6β-methyl-3,20-dioxopregn-4-en-17-yl acetate) has a limit of 1% in the BP, Ph. Int., and EP MPA API monographs as Impurity D and 0.3 % in the BP, Ph. Int., and EP MGA API monographs as impurity F. In the latter case, the drastic difference in impurity limits for the same compound is likely because 6-epimedroxyprogesterone is an epimer of MPA but not MGA, and therefore more difficult to separate.

## 4 Conclusions

For both MPA and MPA injectable products, exposure to a variety of forced degradation conditions generated an array of impurities and degradation products having both known and unknown chemical structures. More impurities and degradation products were generated with acidic, basic, light, and oxidative exposures, but these conditions are less likely under typical storage and transport conditions. Thermal exposures are more likely in the field, but thermal forced degradation conditions yielded little change to the products evaluated. Furthermore, QSAR analysis indicated that the known impurities associated with MPA appear to be no more of a toxicity risk relative to MPA alone. If MPA injectables are exposed to adverse storage conditions, the likelihood of impurities generated is at a minimal risk level, but the toxicity risk of known impurities that may be generated appears limited.

## Declaration of Competing Interest

No competing interests to declare.
