# Supplementary material for: Forced degradation studies of medroxyprogesterone acetate injectable suspensions (150 mg/ml) with implementation of HPLC, mass spectrometry, and QSAR techniques
Source: J Pharm Biomed Anal. 2020 Aug 5;187:113352. doi: 10.1016/j.jpba.2020.113352 (PMC7322552; doi:10.1016/j.jpba.2020.113352)
Supplement: Supplementary file 1 [file mmc1.docx]

**MPA**

**I**

**-**

**-**

**-**

**-**

**-**

**-**

**-**

**-**

**A**

**-**

**-**

**-**

**-**

**B**

**D**

**G**

**Oxidation**

**Base**

**Acid**

**Heat**

**Light**

**Control**
