# Supplementary material for: Forced degradation studies of medroxyprogesterone acetate injectable suspensions (150 mg/ml) with implementation of HPLC, mass spectrometry, and QSAR techniques
Source: J Pharm Biomed Anal. 2020 Aug 5;187:113352. doi: 10.1016/j.jpba.2020.113352 (PMC7322552; doi:10.1016/j.jpba.2020.113352)
Supplement: Supplementary file 4 [file mmc4.docx]

**Group 1 RRT 0.32-0.34**

**Group 2 RRT 0.45-0.46**

**Group 2 RRT 0.52**

**Group 3 RRT 0.49**

**Group 3 RRT 0.55-0.56**
