# Supplementary material for: Forced degradation studies of medroxyprogesterone acetate injectable suspensions (150 mg/ml) with implementation of HPLC, mass spectrometry, and QSAR techniques
Source: J Pharm Biomed Anal. 2020 Aug 5;187:113352. doi: 10.1016/j.jpba.2020.113352 (PMC7322552; doi:10.1016/j.jpba.2020.113352)
Supplement: Supplementary file 5 [file mmc5.docx]

**Supplemental Table 1**. ADMET Predictor^®^ model performance for models used in version 9.0.

| **Model** | **Set** | **Negatives** | **Positives** | **Total** | **Correct** | **Concordance** | **Sensitivity** | **Specificity** |
| --- | --- | --- | --- | --- | --- | --- | --- | --- |
| MUT_98 | Training | 2206 | 86 | 2892 | 2548 | 88.1% | 82.7% | 89.8% |
|  | Test | 568 | 155 | 723 | 621 | 85.9% | 80.0% | 87.5% |
| MUT_m98 | Training | 355 | 148 | 503 | 434 | 86.3% | 80.4% | 88.7% |
|  | Test | 2008 | 836 | 2844 | 2436 | 85.7% | 82.1% | 87.2% |
| MUT_100 | Training | 2378 | 864 | 3242 | 2775 | 85.6% | 82.2% | 86.8% |
|  | Test | 440 | 133 | 573 | 470 | 82.0% | 81.2% | 82.3% |
| MUT_m100 | Training | 351 | 166 | 517 | 426 | 82.4% | 83.1% | 82.1% |
|  | Test | 2011 | 917 | 2928 | 2474 | 84.5% | 81.5% | 85.9% |
| MUT_97+1537 | Training | 1620 | 271 | 1891 | 1690 | 89.4% | 81.2% | 90.7% |
|  | Test | 411 | 62 | 473 | 422 | 89.2% | 83.9% | 90.0% |
| MUT_m97+1537 | Training | 1524 | 244 | 1768 | 1603 | 90.7% | 84.0% | 91.7% |
|  | Test | 378 | 64 | 442 | 393 | 88.9% | 81.3% | 90.2% |
| MUT_1535 | Training | 1538 | 214 | 1752 | 1565 | 89.3% | 85.5% | 89.9% |
|  | Test | 388 | 50 | 438 | 394 | 90.0% | 86.0% | 90.5% |
| MUT_m1535 | Training | 1397 | 245 | 1642 | 1437 | 87.5% | 82.4% | 88.4% |
|  | Test | 366 | 45 | 411 | 351 | 85.4% | 86.7% | 85.2% |
| MUT_102+wp2 | Training | 637 | 166 | 803 | 706 | 87.9% | 81.9% | 89.5% |
|  | Test | 110 | 32 | 142 | 127 | 89.4% | 81.3% | 91.8% |
| MUT_m102+wp2 | Training | 536 | 106 | 642 | 529 | 82.4% | 80.2% | 82.8% |
|  | Test | 143 | 18 | 161 | 132 | 82.0% | 88.9% | 81.1% |
| Chrom_Aberr | Training | 540 | 619 | 1159 | 956 | 82.4% | 83.0% | 81.8% |
|  | Test | 104 | 125 | 229 | 185 | 80.7% | 81.6% | 79.8% |
| Estro_Filter (2D) | Training | 79 | 96 | 175 | 162 | 92.5% | 93.7% | 91.1% |
|  | Test | 19 | 28 | 47 | 43 | 91.4% | 92.8% | 89.4% |
| Andro_Filter | Training | 302 | 218 | 583 | 474 | 81.3% | 81.4% | 81.1% |
|  | Test | 70 | 76 | 146 | 120 | 82.5% | 82.8% | 81.4% |
| Sens_Resp | Training | 144 | 92 | 236 | 211 | 89.4% | 88.0% | 90.2% |
|  | Test | 49 | 25 | 75 | 65 | 87.8% | 84.0% | 89.7% |
| hERG_Filter | Training | 343 | 473 | 816 | 717 | 87.8% | 88.5% | 86.8% |
|  | Test | 76 | 137 | 213 | 184 | 85.8% | 86.9% | 84.0% |
| PLipidosis | Training | 346 | 116 | 462 | 438 | 94.8% | 92.2% | 95.6% |
|  | Test | 83 | 33 | 116 | 110 | 94.8% | 96.9% | 93.9% |
| Ser_AlkPhos | Training | 46 | 59 | 105 | 91 | 86.6% | 89.8% | 82.6% |
|  | Test | 9 | 10 | 19 | 17 | 89.4% | 90.0% | 88.8% |
| Ser_GGT | Training | 66 | 34 | 100 | 95 | 95.0% | 97.0% | 93.9% |
|  | Test | 19 | 7 | 26 | 23 | 88.4% | 85.7% | 89.4% |
| Ser_LDH | Training | 43 | 20 | 63 | 55 | 87.3% | 85.0% | 88.3% |
|  | Test | 11 | 5 | 16 | 13 | 81.2% | 80.0% | 81.8% |
| Ser_AST | Training | 49 | 48 | 97 | 87 | 89.6% | 91.6% | 87.7% |
|  | Test | 15 | 10 | 25 | 20 | 80.0% | 80.0% | 80.0% |
| Ser_ALT | Training | 61 | 83 | 144 | 119 | 82.6% | 84.3% | 80.3% |
|  | Test | 12 | 25 | 37 | 32 | 86.4% | 84.0% | 91.6% |
