# Supplementary material for: Forced degradation studies of medroxyprogesterone acetate injectable suspensions (150 mg/ml) with implementation of HPLC, mass spectrometry, and QSAR techniques
Source: J Pharm Biomed Anal. 2020 Aug 5;187:113352. doi: 10.1016/j.jpba.2020.113352 (PMC7322552; doi:10.1016/j.jpba.2020.113352)
Supplement: Supplementary file 6 [file mmc6.docx]

Supplemental Table 2. ADMET Predictor^®^ results for Ames mutagenicity for MPA and its impurities in models of 5 different strains of *Salmonella typhimurium*, both with metabolic activation (denoted by m notation) and without. Negative sign (-) indicates the compound is predicted to be non-mutagenic in that model.

| Name | CASRN | ID | TOX_MUT_97+1537/ TOX_MUT_m97+1537 | TOX_MUT_98/  TOX_MUT_m98 | TOX_MUT_100/  TOX_MUT_m100 | TOX_MUT_102+wp2/  TOX_MUT_m102+wp2 | TOX_MUT_1535/  TOX_MUT_m1535 |
| --- | --- | --- | --- | --- | --- | --- | --- |
| Medroxyprogesterone Acetate | 71-58-9 | MPA | -/- | -/- | -/- | -/- | -/- |
| 6-Hydroxymedroxyprogesterone Acetate | 984-47-4 | A | -/- | -/- | -/- | -/- | -/- |
| Medroxyprogesterone | 520-85-4 | B | -/- | -/- | -/- | -/- | -/- |
| 6α,17α-Dimethyl-3,17-dioxo-D-homoandrost-4-en-17α-yl Acetate | Not known | C | -/- | -/- | -/- | -/- | -/- |
| 6-Epimedroxyprogesterone Acetate | 2242-65-1 | D | -/- | -/- | -/- | -/- | -/- |
| 6-Methylenehydroxyprogesterone Acetate | 32634-95-0 | E | -/- | -/- | -/- | -/- | -/- |
| 4,5-Dihydromedroxyprogesterone Acetate | 69688-15-9 | F | -/- | -/- | -/- | -/- | -/- |
| Megestrol Acetate | 595-33-5 | G | -/- | -/- | -/- | -/- | -/- |
| Hydroxyprogesterone Acetate | 17308-02-0 | H | -/- | -/- | -/- | -/- | -/- |
| 17β-Hydroxy-6α,17α-dimethyl-D-homoandrost-4-en-3,17-dione | Not known | I | -/- | -/- | -/- | -/- | -/- |
