# Supplementary material for: Forced degradation studies of medroxyprogesterone acetate injectable suspensions (150 mg/ml) with implementation of HPLC, mass spectrometry, and QSAR techniques
Source: J Pharm Biomed Anal. 2020 Aug 5;187:113352. doi: 10.1016/j.jpba.2020.113352 (PMC7322552; doi:10.1016/j.jpba.2020.113352)
Supplement: Supplementary file 7 [file mmc7.docx]

Supplemental Table 3. Toxtree structural alerts for genotoxic and non-genotoxic carcinogenicity for MPA and its impurities.

| Name | CASRN | ID | Structural Alert for Genotoxic Carcinogenicity | Structural Alert for Non-Genotoxic Carcinogenicity |
| --- | --- | --- | --- | --- |
| Medroxyprogesterone Acetate | 71-58-9 | MPA | α,β-unsaturated carbonyl | None |
| 6-Hydroxymedroxyprogesterone Acetate | 984-47-4 | A | α,β-unsaturated carbonyl | None |
| Medroxyprogesterone | 520-85-4 | B | α,β-unsaturated carbonyl | None |
| 6α,17α-Dimethyl-3,17-dioxo-D-homoandrost-4-en-17α-yl Acetate | Not known | C | α,β-unsaturated carbonyl | None |
| 6-Epimedroxyprogesterone Acetate | 2242-65-1 | D | α,β-unsaturated carbonyl | None |
| 6-Methylenehydroxyprogesterone Acetate | 32634-95-0 | E | α,β-unsaturated carbonyl | None |
| 4,5-Dihydromedroxyprogesterone Acetate | 69688-15-9 | F | None | None |
| Megestrol Acetate | 595-33-5 | G | α,β-unsaturated carbonyl | None |
| Hydroxyprogesterone Acetate | 17308-02-0 | H | α,β-unsaturated carbonyl | None |
| 17β-Hydroxy-6α,17α-dimethyl-D-homoandrost-4-en-3,17-dione | Not known | I | α,β-unsaturated carbonyl | None |
